# Risk factors affecting the 1-year outcomes of minor ischemic stroke: results from Xi’an stroke registry study of China

**DOI:** 10.1186/s12883-020-01954-3

**Published:** 2020-10-20

**Authors:** Zhongzhong Liu, Wenjuan Lin, Qingli Lu, Jing Wang, Pei Liu, Xuemei Lin, Fang Wang, Yaling Shi, Qing Wang, Guozheng Liu, Songdi Wu

**Affiliations:** 1 The First Affiliated Hospital of Northwest University, Xi’an, 710069 Shaanxi China; 2 Department of Neurology, The First Hospital of Xi’an, No.30, Fenxiang Road, South Street, Xi’an, 710002 Shaanxi China; 3 College of life Science, Northwest University, Xi’an, 710069 Shaanxi China

**Keywords:** Risk factors, Minor ischemic stroke, Stroke recurrence, Disability, All-cause mortality

## Abstract

**Background:**

The prevalence of stroke recurrence, disability, and all-cause mortality of patients with minor ischemic stroke (MIS) remains problematic. The aim of the present study was to identify risk factors associated with adverse outcomes at 1 year after MIS in the Xi’an region of China.

**Methods:**

This prospective cohort study included MIS patients above 18 years old with National Institutes of Health Stroke Scale (NIHSS) score ≤ 3 who were treated in any of four hospitals in Xi’an region of China between January and December 2015. The 1-year prevalence of stroke recurrence, disability, and all-cause mortality were evaluated, respectively. Multivariate logistic regression analysis was performed to assess the association between the identified risk factors and clinical outcomes.

**Results:**

In this study, 131(10.5%, 131/1252) patients were lost to follow-up at 1 year. A total of 1121 patients were included for analysis, the prevalence of stroke recurrence, disability, and all-cause mortality at 1 year after MIS were 3.4% (38/1121), 9.3% (104/1121), and 3.3% (37/1121), respectively. Multivariate logistic regression analysis identified age, current smoking, and pneumonia as independent risk factors for stroke recurrence. Age, pneumonia, and alkaline phosphatase were independent risk factors for all-cause mortality. Independent risk factors for disability were age, pneumonia, NIHSS score on admission, and leukocyte count.

**Conclusions:**

The 1-year outcomes of MIS in Xi’an region of China were not optimistic, especially with a high prevalence of disability. The present study indicated that age and pneumonia were the common independent risk factors affecting the 1-year outcomes of MIS in Xi’an region of China.

## Background

The prevalence of stroke has significantly increased over the last two decades worldwide [1]. The Global Burden of Disease Study 2010 reported approximately 1.7 million deaths by stroke, which has become the leading cause of death and adult disability in China [2, 3]. The high recurrence and disability rates seriously affect the health and quality of life of stroke patients. A minor ischemic stroke (MIS) is defined as a National Institutes of Health Stroke Scale (NIHSS) score of ≤3 [4]. A MIS is typically treated conservatively with antiplatelet agents and general strategies for the prevention of vascular injury. Yet, the rate of recurrent stroke and progression of stroke remain high, as up to one in four of MIS patients are disabled or died at follow-up [5]. Therefore, it is critical to identify risk factors for stroke recurrence, disability, and all-cause mortality associated with MIS, as most previous studies had focused on long-term risk factors and early detection, while data regarding regional and 1-year risk factors for poor clinical outcomes are relatively limited [6, 7]. Moreover, there may be differences in risk factors for poor clinical outcomes of MIS patients among different countries, regions, and ethnicities. In this study, data regarding the prevalence and poor clinical outcomes of MIS from January to December 2015 were collected from four level 3 first-class hospitals in the Xi’an region of China to identify risk factors associated with stroke recurrence, disability, and all-cause mortality at 1 year after MIS.

## Methods

### Study population

This prospective cohort study included MIS patients aged ≥18 years old who were treated at any of four hospitals in the Xi’an region of China from January to December 2015. Diagnosis of acute ischemic stroke by computed tomography (CT) or magnetic resonance imaging (MRI) according to world Health Organization criteria [8]. The NIHSS score on admission was used to assess the initial neurological severity of the patients [4, 9]. MIS is defined as a NIHSS score at admission ≤3 [4]. Patients diagnosed with MIS due to non-vascular causes (primary and meta-static neoplasms, paralysis after seizure, traumatic brain injury, etc.) that led to a brain function deficit or intracerebral hemorrhage, as determined by CT or MRI, were excluded from analysis, as were those with incomplete follow-up data at 1 year after MIS onset. The diagnostic criteria of all participating hospitals were consistent.

### Data collection

Baseline informations were collected from the four level 3 first-class hospitals participating in this study within 24 h after admission, including demographic information, past medical history, admission evaluation, laboratory data and complications [10]. Peripheral vascular disease (PVD) was defined as intermittent claudication, an ankle brachial index < 0.9, or history of intermittent claudication with relevant interventional therapies (lower limb artery angioplasty/bypass/other vascular interventional treatments/lower limb amputation). Body mass index (BMI) and other associated complications were defined in accordance with the Chinese National Stroke Registry study [6]. The NIHSS score was used to assess the severity of neurological impairment within 24 h of admission [11]. The occurrence of pneumonia during hospitalization was also recorded. All fasting blood samples were processed within 2 h of collection. Measurements of quality control specimens were conducted in a blinded manner in a central laboratory.

### Outcome assessment

The patients were followed up at 1, 3, 6 and 12 months after MIS onset. All enrolled patients were interviewed face-to-face or contacted over the telephone by trained research coordinators. The interviewers were trained centrally with a standardized interview protocol and were blinded to a history of MIS for all patients [12]. The outcome events of 1-year follow-up in this study included stroke recurrence, disability and all-cause mortality. Stroke recurrence was defined as the occurrence of new acute stroke events during follow-up (including cerebral infarction, cerebral hemorrhage, subarachnoid hemorrhage), and the diagnosis of acute stroke were consistent with the World Health Organization definition stroke diagnostic criteria [13]. Confirmation of outcome events were sought from the treating hospital, and suspected stroke recurrence events without hospitalization were judged by independent outcome events judgement committee. Stroke disability was defined as a modified Rankin scale score ≥ 3 (include death) at 1 year after MIS onset [14]. All-cause mortality was defined as death from any cause, as confirmed by either a death certificate from the medical record of the treating hospital or local citizen registry.

### Statistical analysis

Continuous variables that conformed to a normal distribution were expressed as mean ± standard deviation, and did not conform to a normal distribution were expressed as median (interquartile range). Categorical variables are reported as the frequency (%). The chi-squared test (or Fisher’s exact test, where appropriate) was used for comparison between groups, one-way analysis of variance (or Kruskal-Wallis test, where appropriate) for continuous variables. Univariate logistic regression analysis was used to identify baseline differences in clinical variables of patients with vs. without stroke recurrence, disability, and all-cause mortality. Multivariate logistic regression analysis was performed to analyze the associated affect factors of 1-year outcome events of MIS after adjustment for relevant covariates. Sensitive analysis was performed to analyze the influence between the loss to follow up patients and the clinical outcomes. All the estimates parameters were significant at *p* < 0.05 level and the clinical significance were included in the multivariable logistic regression analysis. A two-tailed probability (*p*) value of < 0.05 was considered statistically significant. All analyses were performed with R statistical software (http://www.R-project.org; The R Foundation) and EmpowerStats (http://www.empowerstats.com; X&Y Solutions, Inc., Boston, MA, USA).

## Results

### Patients recruitment

**Fig. 1:**
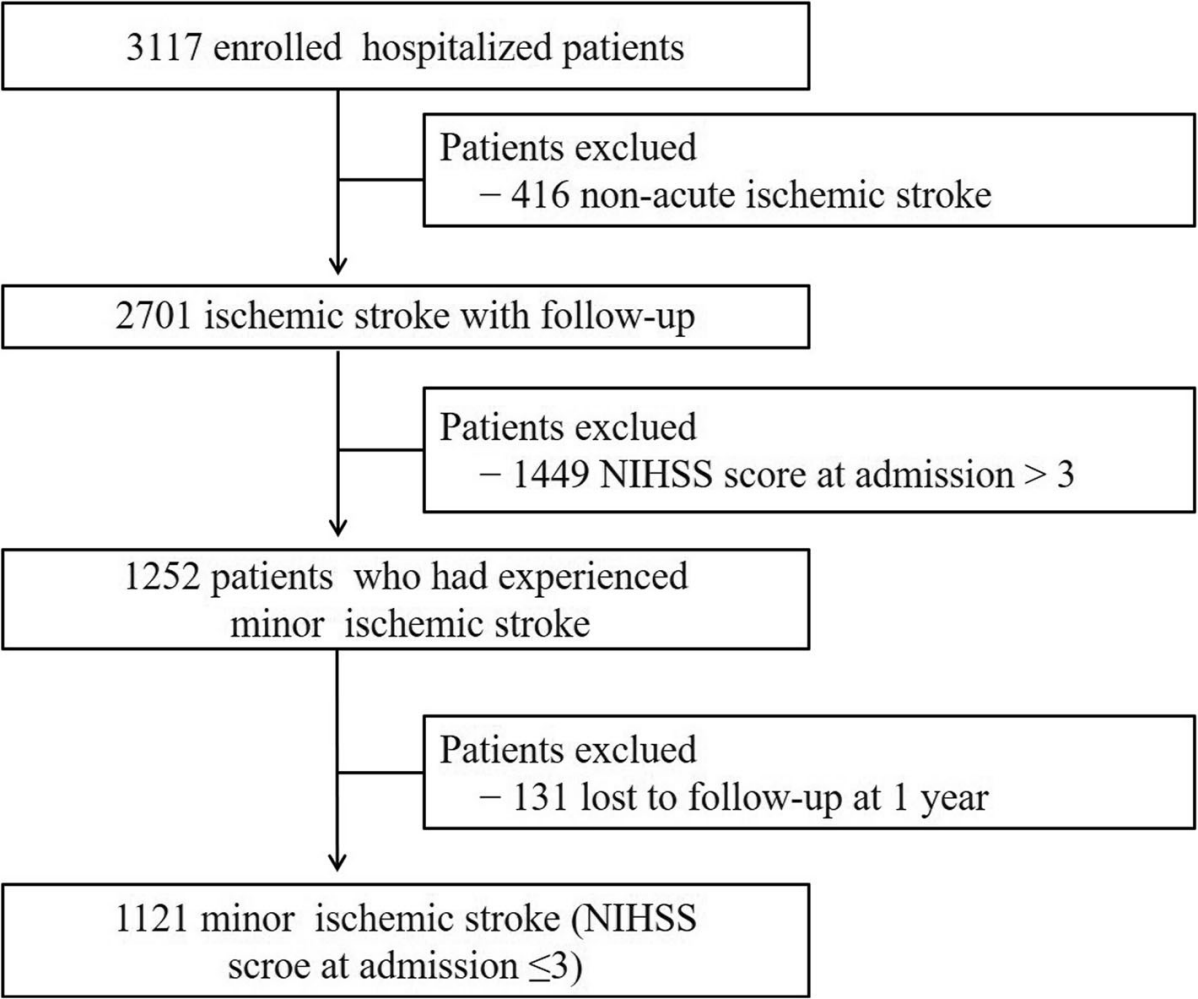
Flow chart showing the patient selection. NIHSS, National Institutes of Health Stroke Scale

Of a total of 3117 patients who were initially enrolled in this study, 416 patients were excluded due to non-acute ischemic stroke in addition to 1449 with an NIHSS score at admission of > 3. One thousand two hundred and fifty-two patients had experienced minor ischemic stroke. Among these, 131(10.5%, 131/1252) patients were lost to follow-up. Finally, a total of 1121 patients with MIS (initial NIHSS score ≤ 3) were included for analysis (Fig. 1).

### Univariate analysis of patients with vs. without stroke recurrence, disability, and all-cause mortality at 1 year after MIS

Among the 1121 patients included for analysis, the prevalence of stroke recurrence, disability, and all-cause mortality at 1 year after MIS was 3.4% (38/1121), 9.3% (104/1121), and 3.3% (37/1121), respectively (Tables 1, 2, and 3). The results of univariate analysis showed that patients with stroke recurrence at 1 year after MIS were more likely to be older, current smokers, and had a history of previous stroke, PVD, and/or pneumonia than those without. There was no significant difference between patients with vs. without stroke recurrence in the sex ratio, BMI on admission, education level, medical insurance type, hypertension, DM, dyslipidemia, AF, moderate to heavy alcohol use, NIHSS score on admission, SBP and DBP on admission, heart rate, total cholesterol, triglycerides, HDL-C, LDL-C, FBG, blood urea nitrogen (BUN), uric acid (UA), alkaline phosphatase (ALP), blood platelet count (BPC), and leukocyte count (Table 1).

**Table 1:** Univariate logistic regression analysis of patients with or without stroke recurrence at 1 year after MIS

Variables	Overall *N* = 1121	Recurrence *N* = 38 (3.4%)	No recurrence *N* = 1083 (96.6%)	OR (95% CI)	*P* value
Demographic information					
Age, median (IQR), year	64.0 (56.0–74.0)	70.0 (61.2–76.8)	64.0 (55.0–73.0)	1.05 (1.02–1.08)	0.001
Male, n(%)	697 (62.2)	29 (76.3)	668 (61.7)	1.97 (0.93–4.17)	0.075
BMI on admission,kg/m^2^	23.9 (22.2–25.6)	24.1 (22.6–26.1)	23.9 (22.1–25.6)	1.00 (0.90–1.10)	0.935
Education level, n(%)					0.068
elementary or below	446 (39.8)	18 (47.4)	428 (39.5)	Ref.	–
middle school	234 (20.9)	6 (15.8)	228 (21.1)	0.62 (0.25–1.57)	0.319
high school or above	441 (39.3)	14 (36.8)	427 (39.4)	0.78 (0.39–1.57)	0.485
Medical insurance type, n(%)					0.636
Urban employees’ medical insurance	591 (52.7)	19 (50.0)	572 (52.8)	Ref.	–
New type rural cooperative medical system	414 (36.9)	17 (44.7)	397 (36.7)	1.29 (0.67–2.48)	0.443
Commercial insurance	2 (0.2)	0 (0.0)	2 (0.2)	0.00 (0.00-∞)	0.996
Out-of-pocket medical	114 (10.2)	2 (5.3)	112 (10.3)	0.55 (0.13–2.35)	0.418
Vascular risk factors					–
Hypertension, n(%)	768 (68.5)	26 (68.4)	742 (68.5)	0.99 (0.50–1.96)	0.974
DM, n(%)	242 (21.6)	12 (31.6)	230 (21.2)	1.68 (0.85–3.32)	0.139
Dyslipidemia, n(%)	77 (6.9)	1 (2.6)	76 (7.0)	0.36 (0.05–2.65)	0.318
AF, n(%)	54 (4.8)	3 (7.9)	51 (4.7)	1.69 (0.52–5.48)	0.386
Smoking, n(%)					0.017
Never smoking	665 (59.3)	12 (31.6)	653 (60.3)	Ref.	–
smoking cessation	169 (15.1)	9 (23.7)	160 (14.8)	1.85 (0.89–4.38)	0.068
Current smoking	287 (25.6)	17 (44.7)	270 (24.9)	1.24 (1.11–2.96)	0.034
Moderate or heavy alcohol use, n(%)	271 (24.2)	7 (18.4)	264 (24.4)	0.70 (0.31–1.59)	0.391
Previous stroke, n(%)	297 (26.5)	18 (47.4)	279 (25.8)	2.54 (1.34–4.80)	0.004
PVD, n(%)	29 (2.6)	3 (7.9)	26 (2.4)	3.35 (1.03–10.91)	0.044
Pneumonia, n(%)	25 (2.2)	6 (15.8)	19 (1.8)	9.89 (4.13–23.68)	0.001
NIHSS score on admission	1.0 (0.0–2.0)	1.5 (0.0–3.0)	1.0 (0.0–2.0)	1.04 (0.80–1.37)	0.755
SBP on admission, mmHg	144.8 ± 20.7	145.6 ± 24.2	144.8 ± 20.6	1.00 (0.99–1.02)	0.788
DBP on admission, mmHg	85.7 ± 12.2	86.6 ± 14.6	85.7 ± 12.1	1.01 (0.98–1.03)	0.661
Heart rate, bmp	74.3 ± 9.4	76.8 ± 10.0	74.2 ± 9.3	1.03 (1.00–1.06)	0.086
Laboratory findings					
Total cholesterol, mmol/L	4.4 ± 1.0	4.5 ± 0.9	4.4 ± 1.0	1.04 (0.76–1.41)	0.821
Triglyceride, mmol/L	1.7 ± 1.2	1.6 ± 0.9	1.7 ± 1.2	0.91 (0.65–1.26)	0.560
HDL-C, mmol/L	1.1 ± 0.3	1.1 ± 0.3	1.1 ± 0.3	0.57 (0.18–1.79)	0.337
LDL-C, mmol/L	2.6 ± 0.8	2.8 ± 0.7	2.6 ± 0.9	1.19 (0.84–1.68)	0.332
FBG, mmol/L	5.8 ± 2.1	6.2 ± 3.0	5.7 ± 2.1	1.09 (0.97–1.22)	0.169
BUN, mmol/L	5.0 ± 1.8	5.3 ± 1.7	5.0 ± 1.8	1.08 (0.93–1.26)	0.321
UA, 10 μmol/L	291.3 ± 96.0	277.9 ± 95.8	291.8 ± 96.1	0.98 (0.95–1.02)	0.387
ALP, U/L	77.6 ± 25.6	83.8 ± 21.5	77.3 ± 25.7	1.01 (1.00–1.02)	0.104
BPC, × 10^11^/L	194.4 ± 58.3	193.4 ± 48.0	194.4 ± 58.6	0.97 (0.55–1.71)	0.906
Leukocyte count, ×10^9^/L	6.7 ± 2.2	7.1 ± 2.5	6.6 ± 2.2	1.08 (0.95–1.21)	0.234

*Abbreviations*: *BMI* Body Mass Index, *DM* Diabetes Mellitus, *AF* Atrial Fibrillation, *PVD* Peripheral Vascular Disease, *NIHSS* National Institutes of Health Stroke Scale, *SBP* Systolic Blood Pressure, *DBP* Diastolic Blood Pressure, *HLD-C* High-Density Lipoprotein Cholesterol, *LDL-C* low-density lipoprotein cholesterol, *FBG* Fasting Blood Glucose, *BUN* Blood Urea Nitrogen, *UA* Uric Acid, *ALP* Alkaline Phosphatase, *BPC* Blood Platelet Count, *CI* confidence internals, *OR* odds ratios, *IQR* interquartile range

**Table 2:** Univariate logistic regression analysis of patients with or without stroke disability at 1 year after MIS

Variables	Overall N = 1121	Disability *N* = 104 (9.3%)	No disability *N* = 1017 (90.7%)	OR (95% CI)	*P* value
Demographic information					
Age, median (IQR), year	64.0 (56.0–74.0)	76.0 (66.0–80.2)	63.0 (55.0–72.0)	1.08 (1.06–1.10)	0.001
Male, n%	697 (62.2)	71 (68.3)	626 (61.6)	1.32 (0.87–1.99)	0.189
BMI on admission, kg/m^2^	23.9 (22.2–25.6)	23.7 (21.6–25.2)	24.0 (22.2–25.6)	0.94 (0.88–1.00)	0.058
Education level, n (%)					0.052
elementary or below	446 (39.8)	52 (50.0)	394 (38.7)	Ref.	–
middle school	234 (20.9)	11 (10.6)	223 (21.9)	0.40 (0.21–1.06)	0.055
high school or above	441 (39.3)	41 (39.4)	400 (39.3)	0.79 (0.52–1.19)	0.254
Medical insurance type, n(%)					0.919
Urban employees’ medical insurance	591 (52.7)	53 (51.0)	538 (52.9)	Ref.	–
New type rural cooperative medical system	414 (36.9)	41 (39.4)	373 (36.7)	1.12 (0.75–1.69)	0.573
Commercial insurance	2 (0.2)	0 (0.0)	2 (0.2)	0.00 (0.00-∞)	0.996
Out-of-pocket medical	114 (10.2)	10 (9.6)	104 (10.2)	0.97 (0.49–1.91)	0.931
Vascular risk factors					
Hypertension, n (%)	768 (68.5)	74 (71.2)	694 (68.2)	1.11 (0.73–1.70)	0.622
DM, n (%)	242 (21.6)	27 (26.0)	215 (21.1)	1.27 (0.82–1.97)	0.289
Dyslipidemia, n (%)	77 (6.9)	6 (5.8)	71 (7.0)	0.81 (0.36–1.86)	0.625
AF, n (%)	54 (4.8)	8 (7.7)	46 (4.5)	1.67 (0.81–3.44)	0.163
Smoking, n (%)					0.053
Never smoking	665 (59.3)	65 (62.5)	600 (59.0)	Ref.	–
Smoking cessation	169 (15.1)	22 (21.2)	147 (14.5)	1.37 (0.85–2.23)	0.198
Current smoking	287 (25.6)	17 (16.3)	270 (26.5)	0.60 (0.35–1.03)	0.063
Moderate or heavy alcohol use, n (%)	271 (24.2)	19 (18.5)	198 (19.5)	0.89 (0.69–1.32)	0.010
Previous stroke, n (%)	297 (26.5)	40 (38.5)	257 (25.3)	1.78 (1.20–2.64)	0.004
PVD, n (%)	29 (2.6)	4 (3.8)	25 (2.5)	1.57 (0.58–4.26)	0.378
Pneumonia, n (%)	25 (2.2)	12 (11.5)	13 (1.3)	7.58 (4.15–13.84)	0.001
NIHSS score on dmission	1.0 (0.0–2.0)	2.0 (0.0–3.0)	1.0 (0.0–2.0)	1.28 (1.08–1.50)	0.004
SBP on admission, mmHg	144.8 ± 20.7	146.8 ± 22.2	144.6 ± 20.6	1.00 (1.00–1.01)	0.297
DBP on admission, mmHg	85.7 ± 12.2	84.7 ± 14.9	85.8 ± 11.9	0.99 (0.98–1.01)	0.390
Heart rate, bmp	74.3 ± 9.4	75.5 ± 9.0	74.1 ± 9.4	1.01 (0.99–1.03)	0.154
Laboratory findings					
Total cholesterol, mmol/L	4.4 ± 1.0	4.4 ± 1.0	4.4 ± 1.0	0.95 (0.78–1.15)	0.598
Triglyceride, mmol/L	1.7 ± 1.2	1.6 ± 1.0	1.7 ± 1.2	0.94 (0.78–1.13)	0.511
HDL-C, mmol/L	1.1 ± 0.3	1.2 ± 0.4	1.1 ± 0.3	1.19 (0.65–2.18)	0.565
LDL-C, mmol/L	2.6 ± 0.8	2.6 ± 0.8	2.6 ± 0.9	0.92 (0.72–1.17)	0.482
FBG, mmol/L	5.8 ± 2.1	6.1 ± 2.7	5.7 ± 2.1	1.07 (0.99–1.16)	0.099
BUN, mmol/L	5.0 ± 1.8	5.7 ± 1.9	4.9 ± 1.8	1.17 (1.08–1.27)	0.001
UA, 10 μmol/L	291.3 ± 96.0	301.6 ± 114.1	290.2 ± 94.0	1.01 (0.99–1.03)	0.296
ALP, U/L	77.6 ± 25.6	80.6 ± 34.5	77.2 ± 24.5	1.01 (1.00–1.01)	0.165
BPC, ×10^11^/L	194.4 ± 58.3	189.7 ± 55.1	194.8 ± 58.6	0.86 (0.60–1.23)	0.386
Leukocyte count, ×10^9^/L	6.7 ± 2.2	7.2 ± 2.8	6.6 ± 2.2	1.09 (1.01–1.17)	0.024

*Abbreviations*: *BMI* Body Mass Index, *DM* Diabetes Mellitus, *AF* Atrial Fibrillation, *PVD* Peripheral Vascular Disease, *NIHSS* National Institutes of Health Stroke Scale, *SBP* Systolic Blood Pressure, *DBP* Diastolic Blood Pressure, *HLD-C* High-Density Lipoprotein Cholesterol, *LDL-C* low-density lipoprotein cholesterol, *FBG* Fasting Blood Glucose, *BUN* Blood Urea Nitrogen, *UA* Uric Acid, *ALP* Alkaline Phosphatase, *BPC* Blood Platelet Count, *CI* confidence internals, *OR* odds ratios, *IQR* interquartile range

**Table 3:** Univariate logistic regression analysis of patients with or without all-cause mortality at 1 year after MIS

Variables	Overall 1121	Death *N* = 37 (3.3%)	No death *N* = 1084 (96.7%)	OR (95% CI)	*P* value
Demographic information					
Age, median (IQR), year	64.0 (56.0–74.0)	75.0 (65.0–80.0)	64.0 (55.0–73.0)	1.07 (1.04–1.11)	0.001
Male, n%	697 (62.2)	28 (75.7)	669 (61.7)	1.91 (0.90–4.04)	0.092
BMI on admission, kg/m^2^	23.9 (22.2–25.6)	23.7 (21.5–24.8)	24.0 (22.2–25.6)	0.93 (0.83–1.04)	0.179
Education level, n (%)					0.494
elementary or below	446 (39.8)	15 (40.5)	431 (39.8)	Ref.	–
middle school	234 (20.9)	5 (13.5)	229 (21.1)	0.62 (0.23–1.71)	0.356
high school or above	441 (39.3)	17 (45.9)	424 (39.1)	1.13 (0.57–2.27)	0.723
Medical insurance type, n (%)					0.944
Urban employees’ medical insurance	591 (52.7)	21 (56.8)	570 (52.6)	Ref.	–
New type rural cooperative medical system	414 (36.9)	13 (35.1)	401 (37.0)	0.90 (0.45–1.79)	0.761
Commercial insurance	2 (0.2)	0 (0.0)	2 (0.2)	0.00 (0.00-∞)	0.997
Out-of-pocket medical	114 (10.2)	3 (8.1)	111 (10.2)	0.74 (0.22–2.47)	0.622
Vascular risk factors					
Hypertension, n (%)	768 (68.5)	22 (59.5)	746 (68.8)	0.66 (0.34–1.28)	0.219
DM, n (%)	242 (21.6)	7 (18.9)	235 (21.7)	0.85 (0.37–1.92)	0.689
Dyslipidemia, n (%)	77 (6.9)	1 (2.7)	76 (7.0)	0.37 (0.05–2.71)	0.328
AF, n (%)	54 (4.8)	4 (10.8)	50 (4.6)	2.39 (0.85–6.75)	0.099
Smoking, n (%)					0.272
Never smoking	665 (59.3)	19 (51.4)	646 (59.6)	Ref.	–
smoking cessation	169 (15.1)	9 (24.3)	160 (14.8)	1.92 (0.87–4.24)	0.107
Current smoking	287 (25.6)	9 (24.3)	278 (25.6)	1.09 (0.50–2.42)	0.823
Moderate or heavy alcohol use, n (%)	271 (24.2)	6 (16.2)	265 (24.4)	0.59 (0.25–1.42)	0.241
Previous stroke, n (%)	297 (26.5)	14 (37.8)	283 (26.1)	1.73 (0.89–3.35)	0.108
PVD, n (%)	29 (2.6)	3 (8.1)	26 (2.4)	3.42 (1.05–11.15)	0.041
Pneumonia, n (%)	25 (2.2)	4 (10.8)	21 (1.9)	6.95 (2.46–19.63)	0.003
NIHSS score on admission	1.0 (0.0–2.0)	2.0 (0.0–3.0)	1.0 (0.0–2.0)	1.25 (0.95–1.65)	0.108
SBP on admission, mmHg	144.8 ± 20.7	146.2 ± 21.2	144.8 ± 20.7	1.00 (0.99–1.02)	0.652
DBP on admission, mmHg	85.7 ± 12.2	85.3 ± 13.7	85.7 ± 12.1	1.00 (0.97–1.02)	0.853
Heart rate, bmp	74.3 ± 9.4	77.0 ± 7.9	74.2 ± 9.4	1.03 (1.00–1.06)	0.061
Laboratory findings					
Total cholesterol, mmol/L	4.4 ± 1.0	4.2 ± 0.7	4.4 ± 1.0	0.79 (0.56–1.12)	0.191
Triglyceride, mmol/L	1.7 ± 1.2	1.4 ± 0.9	1.7 ± 1.2	0.75 (0.50–1.14)	0.180
HDL-C, mmol/L	1.1 ± 0.3	1.1 ± 0.3	1.1 ± 0.3	0.96 (0.33–2.76)	0.939
LDL-C, mmol/L	2.6 ± 0.8	2.4 ± 0.6	2.6 ± 0.9	0.72 (0.47–1.10)	0.132
FBG, mmol/L	5.8 ± 2.1	5.8 ± 2.3	5.8 ± 2.1	1.01 (0.87–1.17)	0.909
BUN, mmol/L	5.0 ± 1.8	5.4 ± 1.6	5.0 ± 1.8	1.11 (0.95–1.29)	0.196
UA, 10 μmol/L	291.3 ± 96.0	319.1 ± 130.8	290.3 ± 94.5	1.03 (1.00–1.06)	0.082
ALP, U/L	77.6 ± 25.6	87.2 ± 43.7	77.2 ± 24.7	1.01 (1.00–1.02)	0.012
BPC, ×10^11^/L	194.4 ± 58.3	181.6 ± 54.1	194.8 ± 58.4	0.67 (0.37–1.22)	0.187
LC, ×10^9^/L	6.7 ± 2.2	6.5 ± 2.3	6.7 ± 2.2	0.97 (0.83–1.13)	0.699

*Abbreviations*: *BMI* Body Mass Index, *DM* Diabetes Mellitus, *AF* Atrial Fibrillation, *PVD* Peripheral Vascular Disease, *NIHSS* National Institutes of Health Stroke Scale, *SBP* Systolic Blood Pressure, *DBP* Diastolic Blood Pressure, *HLD-C* High-Density Lipoprotein Cholesterol, *LDL-C* low-density lipoprotein cholesterol, *FBG* Fasting Blood Glucose, *BUN* Blood Urea Nitrogen, *UA* Uric Acid, *ALP* Alkaline Phosphatase, *BPC* Blood Platelet Count, *CI* confidence internals, *OR* odds ratios, *IQR* interquartile range

As shown in Table 2, older patients were more likely to be disabled at 1 year after MIS (*p* < 0.05) and those with a disability were significantly more likely to have a history of previous stroke and/or pneumonia, and a higher NIHSS score on admission, as well as higher BUN measurements and leukocyte count, as compared to those without. There was no significant difference between patients with vs. without disability in the sex ratio, BMI on admission, education level, medical insurance type, hypertension, DM, dyslipidemia, AF, smoking, moderate to heavy alcohol use, PVD, SBP and DBP on admission, heart rate, total cholesterol, triglyceride, HDL-C, LDL-C, FBG, UA, ALP, and BPC.

Factors associated with all-cause mortality in patients after MIS are shown in Table 3. The results of univariate analysis showed that the factors associated with death included age, PVD, pneumonia, and ALP (all *p* < 0.05).

Regardless of whether the patients lost to follow-up were all considered to have no stroke recurrence, disability or all-cause mortality respectively, sensitivity analysis showed that the risk factors between the two groups with or without this part of patients were almost the same. There were only a few differences between the two groups when the patients lost to follow-up were all considered to be stroke recurrence, disability or all-cause mortality, respectively. In addition, by comparing the clinical characteristics of two groups with or without patients lost to follow-up, only medical insurance type differed between the two groups and there was no statistical difference in other variables (data not shown).

### Risk factors for outcomes at 1 year after MIS

Multivariate logistic regression analysis was used to identify independent risk factors for stroke recurrence, disability, and all-cause mortality at 1 year after MIS. The results showed that age (odds ratio [OR] = 1.04; 95% confidence interval [CI] = 1.01–1.08; *p* = 0.013), current smoking (OR = 3.25; 95% CI = 1.33–7.93; *p* = 0.010), and pneumonia (OR = 6.80; 95% CI = 2.24–20.64; *p* = 0.001) were independent risk factors for stroke recurrence at 1 year after MIS onset. The independent risk factors for stroke disability at 1 year were age (OR = 1.09; 95% CI = 1.06–1.12; *p* = 0.001), pneumonia (OR = 6.24; 95% CI = 2.35–16.55; *p* = 0.000), NIHSS score on admission (OR = 1.24; 95% CI = 1.01–1.53; *p* = 0.036), and leukocyte count (OR = 1.09; 95% CI = 1.00–1.20; *p* = 0.048). Age (OR = 1.06; 95% CI = 1.03–1.11; *p* = 0.001), pneumonia (OR = 4.48; 95% CI = 1.27–15.83; *p* = 0.020), and ALP levels (OR = 1.01; 95% CI = 1.00–1.02; *p* = 0.023) were independent risk factors associated with all-cause mortality at 1 year after MIS onset (Table 4).

**Table 4:** Multivariable logistic regression analysis of independent risk factors associated with stroke recurrence, disability and all-cause mortality at 1 year after MIS onset

Variables	Stroke recurrence	*P* value	Stroke disability	*P* value	Death	*P* value
	OR (95% CI)		OR (95% CI)		OR (95% CI)	
Age	1.04 (1.01–1.08)	0.013	1.09 (1.06–1.12)	0.001	1.06 (1.03–1.11)	0.001
Moderate or heavy Alcohol use	0.36 (0.13–1.03)	0.056	0.45 (0.19–1.05)	0.064	0.31 (0.22–1.00)	0.055
Current smoking	3.25 (1.33–7.93)	0.010	–	–	2.06 (0.78–5.45)	0.146
Previous stroke	1.89 (0.91–3.91)	0.087	1.28 (0.76–2.17)	0.356	–	–
PVD	1.80 (0.37–8.77)	0.465	0.66 (0.14–3.14)	0.601	2.20 (0.44–10.88)	0.334
Pneumonia	6.80 (2.24–20.64)	0.001	6.24 (2.35–16.55)	< 0.001	4.48 (1.27–15.83)	0.020
ALP	–	–	–	–	1.01 (1.00–1.02)	0.023
NIHSS score on admission	0.88 (0.65–1.20)	0.420	1.24 (1.01–1.53)	0.036	1.08 (0.80–1.48)	0.607
BUN	–	–	1.11 (0.99–1.24)	0.088	–	–
Leukocyte count	1.03 (0.90–1.19)	0.651	1.09 (1.00–1.20)	0.048	0.94 (0.80–1.11)	0.465

Stroke disability was defined as modified Rankin Scale≥3. “−“not included in the multivariate analysis

*Abbreviations*: *OR* Odd Ratio, *CI* Confidence Internal, *PVD* Peripheral vascular disease, *ALP* Alkaline phosphatase, *BUN* Blood urea nitrogen; Adjusted for age, education level, moderate or heavy alcohol, current smoking, previous stroke, *PVD,* Pneumonia, *ALP,* NIHSS score on admission, BUN and Leukocyte count

## Discussion

This study is the largest stroke registration study to date in Xi’an, China. The results of the present study showed that a small number of patients with MIS experienced stroke recurrence, disability and all-cause mortality during the 1-year follow-up period. Nevertheless, these results are not optimistic. Hence, the risk factors associated with poor clinical outcomes at 1 year after MIS need to be further investigated. The results suggested that the risk factors associated with poor outcomes at 1 year after MIS stroke (i.e., recurrence, disability, and all-cause mortality) were not entirely consistent in Xi’an, China. Therefore, clinicians should apply early prevention strategies on an individual basis.The results further showed that the prevalence of stroke recurrence at 1 year after MIS in the Xi’an region of China was 3.4%, which was lower than the prevalence of 13.2% at 1 year and that of 9.8% at 3 months reported by the China National Stroke Registry (CNSR) study [6, 15]; lower than the stroke recurrence prevalence of 7.6% in the Clopidogrel in high-risk patients with the Acute Non-disabling Cerebrovascular Events (CHANCE) study [16]; also lower than the stroke recurrence prevalence of 6.1% by analysis of the Korean Multicenter Stroke Registry [17]; but close to the stroke recurrence prevalence of 3.7% recently reported by the TIAregistry.org project [18]. Besides, our data also revealed lower mortality (3.3% vs. 6.3%) and disability (9.3% vs. 17%) at 1 year as compared with the CNSR study [13], but with a comparable mortality (3.3% vs. 4.1%) to the Korean Multicenter Stroke Registry study at 1 year after MIS [17]. These results suggested that the clinical outcomes of patients with MIS may differ among countries and regions. In addition to the differences in study designs, the prevalence of clinical outcomes may also be related to geographical environments, daily habits, economic status, and disease prevention measures, indicating the importance of studies of regional stroke registries.

There are several potential reasons for the lower prevalence of stroke recurrence, disability, and all-cause mortality at 1 year in the present study. First, there were notable differences in the clinical characteristics between our study and previous studies. As compared to the CNSR study [6], patients included in the present study had lower prevalence of hypertension (68.5% vs. 73.6%), DM (21.6% vs. 27.3%), dyslipidemia (6.9% vs. 11.8%), atrial fibrillation (4.8% vs. 5.8%), and previous stroke (26.5% vs. 31.1%), as well as lower NIHSS scores on admission (median, 1 vs. 2). Hence, the prevalence of risk factors for clinical outcomes of stroke in this region was relatively lower, which may be related to the better preventive measures and lifestyles in the Xi’an region, as compared with other regions. Second, differences in study designs and regions may have led to the differences in results. The CNSR study was a nationwide survey [6, 15] and, thus, did not represent the status quo. The datas assessed in the present study were collected from four tertiary grade A hospitals in the Xi’an region, which corresponding to the lower valley of the Wei River in the Guanzhong Plain in northern China. The relatively lower prevalence of poor outcomes may be due to more standardized regimens for the diagnosis, treatment, and prevention of secondary stroke than those in the CNSR study, which included primary, secondary, and tertiary hospitals. Other potential reasons for the lower prevalence of poor outcomes in the present study might be that most of the patients resided in urban areas of the Xi’an region and more than 90% had medical insurance.

Risk factors affecting the 1-year outcomes (i.e., stroke recurrence, disability, and all-cause mortality) after MIS in the Xi’an region of China were investigated. In the present and previous studies, age was identified as an independent risk factor for stroke recurrence, disability, and all-cause mortality at 1 year after MIS [19, 20]. Hence, older patients should be closely monitored for various indicators and early detection and treatment.

In addition, pneumonia was identified as an independent risk factor for stroke recurrence, disability, and all-cause mortality at 1 year after MIS in the Xi’an region, similar with the findings of previous studies [21, 22]. Pneumonia is closely related to dysphagia caused by stroke [22], suggesting that treatment for swallow difficulties after stroke must be improved in the Xi’an region. So, clinicians should promptly evaluate patients with dysphagia and initiate swallow rehabilitation, dietary guidance, and education of dysphagia in order to reduce the prevalence of pneumonia after MIS and improve treatment outcomes.

Similar to previous studies [23–25], current smoking was found to be an independent risk factor associated with 1-year stroke recurrence after MIS. After stroke, persistent smoking increases the risk of stroke recurrence. There exists a dose-response relationship between smoking quantity and the risk of stroke recurrence [24, 25] because smoking increases the short-term risk of stroke by promoting thrombosis and reducing cerebral blood flow via arterial vasoconstriction [26, 27]. Therefore, it is important to control smoking among MIS patients.

In this study, an elevation in ALP levels was an independent risk factor for all-cause mortality, in accordance with the findings of previous studies [28–30]. Elevated ALP was related with an increased risk of all-cause mortality in patients with end-stage renal disease [28, 29] and as an independent predictor of poor outcomes of patients with preserved kidney function in the CNSR study [30]. As a possible explanation, serum ALP has been implicated in the pathogenesis of vascular calcification and subclinical atherosclerosis [31, 32]. Vascular calcification plays a significant role in the process of atherosclerosis and also leads to increase vascular stiffness and reduce vascular compliance. So, clinicians should pay more attention to ALP levels in patients with MIS, as early detection and intervention may reduce the risk of death within 1 year after stroke.

In the present study, the NIHSS score and leukocyte count on admission were identified as risk factors for stroke disability at 1 year after MIS, which was consistent with the findings of previous results [33–36]. A higher NIHSS score indicates severe neurological impairment. Because there is no specific treatment for cerebral function injury caused by stroke, the outcomes of the majority of patients with severe neurological impairment were generally poor. Previous studies have reported that a high leukocyte count in the early stage of stroke was closely related to the severity of stroke and co-infection, which led to aggravation of stroke and subsequent disability [37, 38]. However, an elevated leukocyte count in the early stage of stroke may not necessarily be caused by infection, thus the clinician should assess the presence of co-infections. For non-infectious stroke, the patient’s family members should be informed of a potentially poor outcome as early as possible. Early prevention and treatment of digestive tract ulcers and acute brain-heart syndrome may be hampered by a state of stress.

In addition, multivariate analysis showed that alcohol seemed to be a protective factor. The possible explanation is that we only recorded whether the alcohol used or not, but did not record the amount of alcohol consumed. However, based on the clinical characteristics, the mean age was higher in the patients with MIS who had adverse outcomes (include stroke recurrence, disability, all-cause mortality) at 1-year follow up. This may be due to the fact that most of the patients were in good health before the onset, more patients may have previously drunk alcohol. This phenomenon may lead to the tendency of alcohol consumption to be a protective factor in our multivariate analysis, but the result was not statistically significant.

There were some limitations to this study that should be addressed. First, the four hospitals participating in this study were not selected at random, thus there was potential for selection bias when evaluated the real burden of the disease in the Xi’an region of China. In addition, all the participating hospitals were level 3 first-class hospitals that may not necessarily represent the status quo of MIS treatment in community hospitals. Second, the focus of this study was the influence of risk factors on admission and during hospitalization on 1-year outcomes, so potential factors after discharge were not analyzed. Third, the data obtained from cerebrovascular and neurological imaging in this study were incomplete, so there were a lack of image-related risk factors, such as infarct volume and infarct location. Forth, in this study, 131 (10.5%) patients were lost to follow-up at 1 year after MIS. However, the sensitivity analysis showed that the patients lost to follow-up in this study were nearly random, which did not affect the results.

## Conclusions

In conclusion, the 1-year outcomes of MIS in Xi’an region of China were not optimistic, especially high prevalence of disability. The present study indicated several risk factors affecting the 1-year outcomes of MIS in Xi’an region of China. Of note, age and pneumonia were the common independent risk factors for 1-year stroke recurrence, disability, and all-cause mortality.

## Data Availability

The datasets used and/or analyzed during the current study are available from the corresponding author on reasonable request.

## Abbreviations

AF: atrial fibrillation
ALP: alkaline phosphatase
BMI: body mass index
BPC: blood platelet count
BUN: blood urea nitrogen
CHANCE: clopidogrel in high-risk patients with acute non-disabling cerebrovascular events
CI: confidence internal
CNSR: China national stroke registry
CT: computed tomography
DBP: diastolic blood pressure
DM: diabetes mellitus
FBG: fasting blood glucose
HLD-C: high-density lipoprotein cholesterol
IQR: interquartile range
LDL-C: low-density lipoprotein cholesterol
MIS: minor ischemic stroke
MRI: magnetic resonance imaging
NIHSS: National Institutes of Health stroke scale
OR: odds ratio
PVD: peripheral vascular disease
SBP: systolic blood pressure
UA: uric acid
